# Composition Driven Redistribution of Feeding Mechanisms in Hypoeutectic Al–Si–Mg Alloys

**DOI:** 10.3390/ma19091744

**Published:** 2026-04-24

**Authors:** Aleksandra Patarić, Mile Djurdjevic, Srecko Manasijevic, Srecko Stopic, Marija Mihailović

**Affiliations:** 1 Institute of Chemistry, Technology and Metallurgy, National Institute of the Republic of Serbia, University of Belgrade, Njegoševa 12, 11000 Belgrade, Serbia; aleksandra.pataric@ihtm.bg.ac.rs; 2 Department of Material Science and Technology, University of Applied Sciences Upper Austria, Roseggerstraße 15, 4600 Wels, Austria; djurdjevicmile506@gmail.com; 3 Lola Institute, Kneza Višeslava 70a, 11030 Belgrade, Serbia; srecko.manasijevic@li.rs; 4 IME Process Metallurgy and Metal Recycling, RWTH Aachen University, Intzestrasse 3, 52056 Aachen, Germany; sstopic@metallurgie.rwth-aachen.de

**Keywords:** Al-Si–Mg alloys, thermal analysis, cooling curves, feeding efficiency

## Abstract

This study provides a systematic quantitative assessment of the influence of silicon (5–9 wt.%) and magnesium (0–0.6 wt.%) on the solidification behavior and feeding mechanisms of hypoeutectic Al–Si–Mg casting alloys. Cooling curve analysis combined with first-derivative and Δ*T* (*T*w − *T*c) evaluation was used to determine characteristic solidification temperatures, including the liquidus, dendrite coherency, rigidity, and solidus temperatures, enabling the precise delineation of feeding regions. Increasing silicon content reduced all characteristic temperatures, while magnesium addition exerted a more pronounced effect on rigidity and solidus temperatures, significantly redistributing the relative contributions of mass, interdendritic, and burst feeding. In particular, magnesium addition systematically expanded the interdendritic feeding interval and reduced the burst-feeding range, promoting earlier dendrite interlocking and restricting melt flow during late-stage solidification. Quantitative temperature ratio analysis revealed that magnesium plays a dominant role in shifting feeding toward interdendritic-controlled flow, especially in low-Si alloys. Sand Hourglass testing confirmed that this redistribution directly correlated with increased shrinkage porosity, with the highest porosity observed in the AlSi9Mg0.6 alloy. The results establish a quantitative link between alloy composition, feeding redistribution, and porosity susceptibility, providing a practical framework for optimizing the design and casting performance of Al–Si–Mg alloys.

## 1. Introduction

The growing demand for high-performance Al–Si–Mg casting alloys is driven by their excellent mechanical and physical properties, which make them highly suitable for demanding applications. As a result, these alloys are widely employed in the automotive, aerospace, and general engineering industries, where lightweight, reliable materials are essential. Among them, hypoeutectic Al–Si–Mg alloys are particularly attractive due to a combination of advantageous characteristics, including high fluidity during casting, relatively low thermal expansion, good wear resistance, and strong corrosion resistance.

The overall performance of Al–Si–Mg alloys is largely governed by their chemical composition, with silicon and magnesium playing the most significant roles in determining microstructure and mechanical properties. Silicon improves castability by increasing melt fluidity and reducing the tendency toward hot cracking, while also enhancing wear resistance. Magnesium, on the other hand, enables precipitation hardening, which significantly increases strength, hardness, and fatigue resistance [1,2]. The interaction between these alloying elements directly affects the morphology of primary aluminum and eutectic silicon phases, which, in turn, influences mechanical behavior. Despite these advantages, Al–Si–Mg casting alloys still face challenges during the solidification process, particularly the formation of shrinkage porosity [3,4]. This defect arises from volume contraction during the metal’s transition from liquid to solid and can negatively affect the mechanical properties and component reliability. Therefore, a thorough understanding of solidification behavior and feeding mechanisms is essential for producing defect-free castings. Solidification feeding regions are influenced by several interrelated factors, including alloy composition, solidification range, cooling rate, and casting geometry. Despite literature references on the individual effects of magnesium and/or silicon on feeding behavior [5,6,7,8,9], systematic data on the influence of their combined presence in the chemical composition remain limited. Accordingly, the research presented in this paper constitutes an original contribution, providing a comprehensive and systematic evaluation of the coupled effects of these elements on feeding performance and representing a novel finding to the field. Optimizing the chemical content of both magnesium and silicon through proper alloy design and casting process control is crucial to minimizing porosity and ensuring consistent, high-quality cast components. Alloy solidification was analyzed following Campbell’s [10,11] classification of feeding regions, which includes liquid feeding, mass feeding, interdendritic feeding, burst feeding, and solid feeding. The five feeding mechanisms can be physically interpreted in terms of the dominant flow regime during solidification. Liquid and mass feeding occur under convective flow conditions governed by classical fluid mechanics. In contrast, interdendritic and burst feeding occur after dendrite coherency is reached, when liquid flow is restricted to porous dendritic networks and can be described by Darcy-type filtration. Once the rigidity point is reached, permeability is lost, and feeding becomes blocked, indicating solid feeding conditions. Accurate identification, thorough understanding, and precise quantification of these feeding regions during the solidification of cast Al–Si–Mg alloys are essential for the implementation of effective feeding strategies and improving casting quality. Thermal analysis (TA), combined with first-derivative and Δ*T* (*T*w − *T*c) curve methods, was employed to determine key solidification points, including the liquidus and solidus, as well as dendrite coherency and rigidity points [12,13,14]. The parameter *T*w represents the cooling curve measured near the mold wall, where heat extraction is more intensive due to direct contact with the mold. In contrast, *T*c denotes the cooling curve measured at the center of the sample, where the cooling rate is lower. These boundary temperatures allowed for precise delineation of feeding regions and facilitated both qualitative and quantitative evaluation. Despite advances in the literature [15,16], the systematic quantification of feeding regions remains largely unresolved. The influence of varying silicon contents (5, 7, and 9 wt.%) and varying magnesium contents (0 to 0.6 wt.% in increments of 0.2 wt.%) on the feeding behavior of the alloys was systematically examined, providing a basis for minimizing defect formation and enhancing casting quality. The results offer a novel, integrated perspective on how chemical composition and solidification dynamics jointly affect feeding efficiency in Al–Si–Mg castings. Unlike most previous studies that examined the effects of silicon and magnesium on individual solidification parameters, this work provides a systematic quantitative assessment of how these elements redistribute feeding mechanisms during solidification and how this redistribution correlates with shrinkage porosity formation. The aim of this study is therefore to investigate the influence of silicon and magnesium on the solidification behavior and feeding effectiveness of hypoeutectic Al–Si–Mg alloys. The novelty of this work lies in the quantitative delineation of all feeding regions based on thermal analysis and in establishing a direct link between their redistribution and porosity formation as a function of alloy composition.

## 2. Materials and Methods

The experimental setup focused on keeping all testing conditions constant while varying only the alloy composition. This strategy minimized the influence of known factors such as gas content, oxide concentration, melt nucleation condition, and mold filling behavior, ensuring consistent low-turbulence casting conditions. Emphasis was placed on achieving and maintaining low gas content while ensuring consistently minimal oxide skin formation. All melts were prepared without trace elements such as Sr, Na, Sb, Ti, P, B, Pb, and Sn that could potentially alter the characteristic solidification temperatures of the investigated alloys. The following high-purity raw materials were used in the study: aluminum (commercial purity 99.7 wt.%, TRIMET Aluminium SE, Aluminiumallee 1, D-45356 Essen, Germany); silicon (commercial purity 99.9 wt.%, HOESCH Metallurgie GmbH, Neue Str. 21, 52382 Niederzier, Germany); magnesium (commercial purity 99.9 wt.%, HOESCH Metallurgie GmbH, Neue Str. 21, 52382 Niederzier, Germany).

All alloys were melted in an electric resistance furnace (Nabertherm, Models K 1/10 and KC 2/15) with a 10-kg capacity (Zeller GmbH, Industriestr. 1, A-6845 Hohenems, Austria). The pouring temperature and mold preheating conditions were kept constant for all experiments to ensure comparable thermal conditions and to isolate the effect of alloy composition. While variations in process parameters and casting methods can influence shrinkage behavior, the present study focused on composition-driven effects under controlled conditions. No grain refining agents or modifiers were added to any of the melts. The exclusion of grain refining agents and modifiers was intentional to isolate the effect of alloy composition on solidification behavior and feeding mechanisms. While this approach differs from typical industrial practice, it enables a clearer interpretation of composition-driven effects without interference from additional variables. Therefore, the results of this study should be considered a baseline for understanding more complex systems that include such additions. To investigate the influence of silicon and magnesium as the major alloying elements of AlSi(5, 7, 9)Mg(0, 0.2, 0.4, 0.6) hypoeutectic alloys, on feeding behavior, specific alloy compositions were prepared as detailed in Table 1. Table 1 shows that there were three series of Al alloys with silicon contents of 5, 7, and 9%. In each series, the magnesium content varied from 0 to 0.6% in increments of 0.2%. The chemical composition of all tested alloys was examined through optical emission spectroscopy (OES) analysis (Type Spectrolab, SPECTRO Analytical Instruments GmbH, Boschstr. 10, 47533 Kleve, Germany).

### 2.1. Sand Hourglass Test

The Sand Hourglass technological test, developed in accordance with Huebler’s methodology [17], enables the evaluation of feeding behavior under realistic processing conditions.

**Figure 1:**
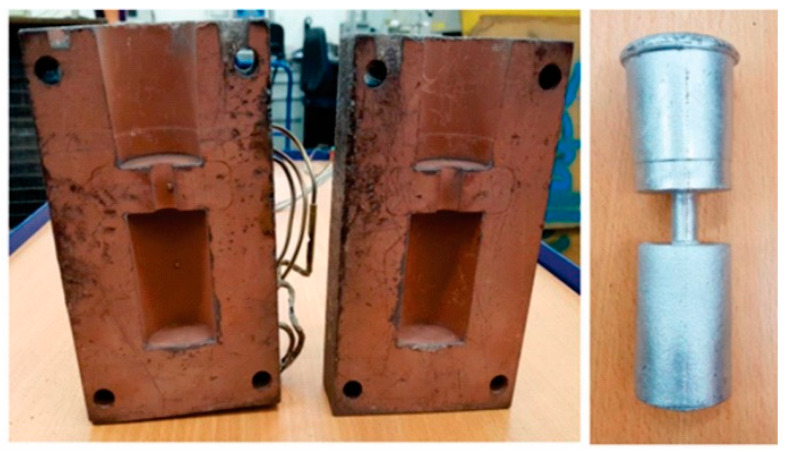
“Sand Hourglass” mold (**left**) and sample (**right**) [18].

The test sample comprised two geometrically identical cylindrical specimens connected by a feeder neck, as illustrated in Figure 1. The upper specimen functioned as the feeder, while the lower specimen represented the casting. The feeder is designed to compensate for solidification shrinkage and to promote directional solidification toward the upper part of the sample. Therefore, porosity evaluation was performed exclusively on the lower specimen, which represents the region most sensitive to feeding limitations. The Sand Hourglass mold features two chambers, each with a diameter of approximately 40 mm and a height of 45 mm, interconnected by a central feeder neck measuring 15–20 mm in diameter and 20–25 mm in length. Each specimen had a volume of approximately 55 cm^3^ and a mass of about 150 g. The feeder neck was specifically dimensioned to ensure complete feeding of the casting under optimal conditions.

As feeding efficiency decreases due to variations in solidification morphology, macro- and micro-shrinkage pores or other casting defects may develop in the lower specimen. The feeding performance was assessed by separating the feeder from the casting at the central interface and applying standard metallographic procedures.

Individual samples for each alloy composition listed in Table 1 were cast using the Sand Hourglass mold. The casting was performed at a pouring temperature of 750 °C, with the mold preheated to 300 °C.

The evaluation was conducted exclusively on the lower portion of the Sand Hourglass sample (the “casting”). This section was bisected and sequentially ground using abrasive wheels of increasing fineness (#80, #320, #500, and #1200). Following preparation, the samples were documented photographically and examined under a stereo microscope. Porosity measurements were performed using image analysis, enabling quantitative comparisons across the different alloy compositions.

### 2.2. Thermal Analysis Procedure

Thermal analysis was performed using standardized test samples weighing 200 ± 10 g for each alloy composition listed in Table 1. The samples were placed in conical steel crucibles with a height of 60 mm, a top diameter of 55 mm, and a bottom diameter of 45 mm, with each crucible weighing 50 g.

Two calibrated K-type thermocouples (OMEGA Engineering, Daimlerstrasse 26, 75392 Deckenpfronn, Germany), each with an accuracy of ±0.10 °C, were placed within each crucible: one positioned 20 mm above the crucible bottom near the wall, and the other at the center of the test sample. Temperature data were recorded continuously over the cooling range from 700 °C to 400 °C.

Thermal data were acquired using a high-speed National Instruments data acquisition system (Type NI cDAQ-9171, National Instruments, 11,500 N. Mopac Expwy, Austin, TX 78759-3504, USA) connected to a personal computer, which captured five data points per second during all experimental trials. Cooling conditions were kept constant throughout all experiments, with an average cooling rate of approximately 10 °C/min. The cooling rate was determined from the temperature data recorded by the thermocouple at the center of the sample (*T*c) by calculating the ratio of the temperature difference between the liquidus and solidus temperatures to the corresponding solidification time interval between them. Due to heat losses during melt transfer and pouring into the crucible, the maximum temperatures recorded at the start of thermal analysis were lower than the pouring temperature and ranged from approximately 630 °C to 700 °C. These variations did not affect the evaluation of the solidification parameters, as all characteristic temperatures and cooling rates were determined relative to the liquidus–solidus interval for each sample. All experiments were performed in duplicate to ensure reproducibility of the results. The observed variations between repeated measurements were minor and did not affect the identified trends.

## 3. Results and Discussion

The most predominant and critical defect in aluminum castings is shrinkage porosity, which primarily originates from insufficient feeding or hydrogen precipitation during solidification. These defects result in substantial scrap losses and limit the use of aluminum castings in critical structural components. Therefore, understanding the relationship between alloy composition, solidification behavior, and feeding efficiency is essential for improving casting quality. The present study focused on quantifying the influence of silicon and magnesium on characteristic solidification temperatures and the associated feeding mechanisms in hypoeutectic Al–Si–Mg alloys. Characteristic temperatures were determined using cooling curve analysis, together with first derivative and Δ*T* (*T*w − *T*c) analysis.

**Figure 2:**
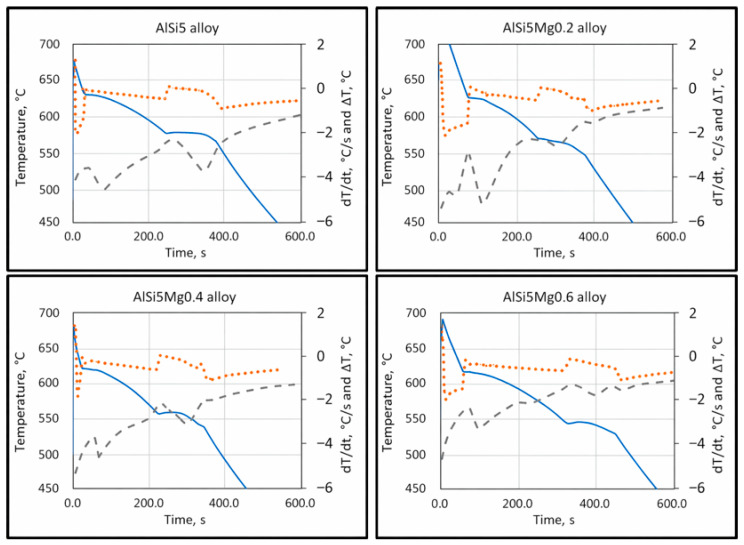
Cooling curves (full line), first derivative curves (dotted line), and delta T curves (interrupted line) of AlSi5, AlSi5Mg0.2, AlSi5Mg0.4, and AlSi5Mg0.6 hypoeutectic cast alloys.

**Figure 3:**
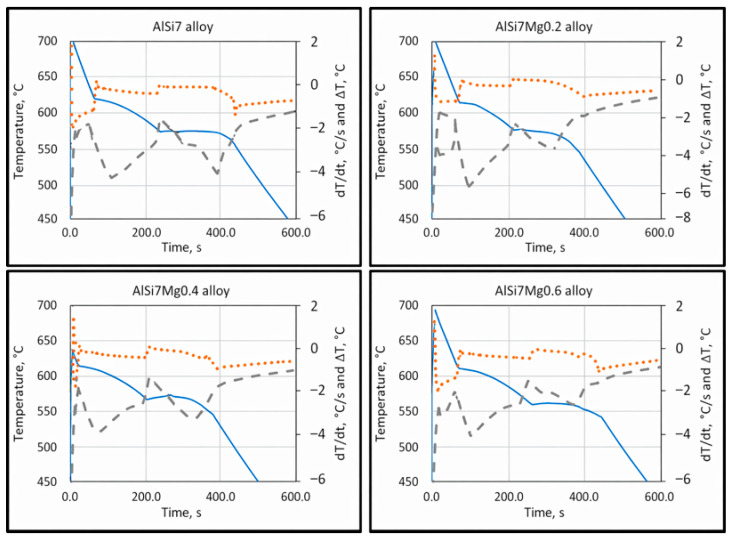
Cooling curves (full line), first derivative curves (dotted line), and delta T curves (interrupted line) of AlSi7, AlSi7Mg0.2, AlSi7Mg0.4, and AlSi7Mg0.6 hypoeutectic cast alloys [18].

**Figure 4:**
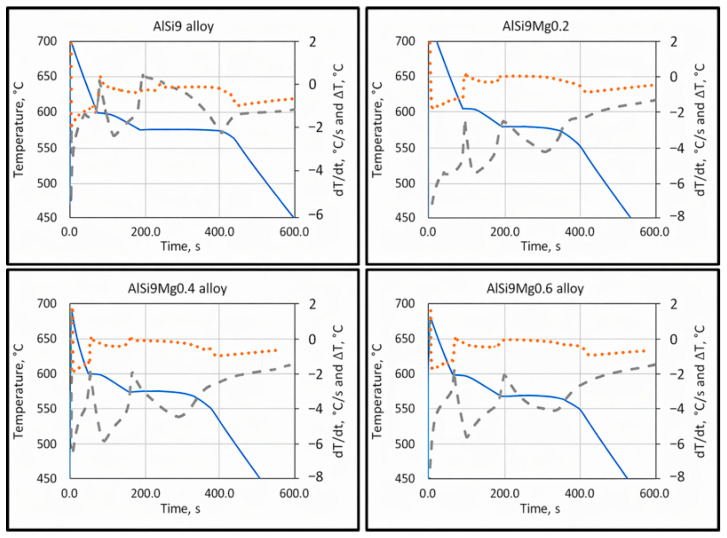
Cooling curves (full line), first derivative curves (dotted line), and delta T curves (interrupted line) of AlSi9, AlSi9Mg0.2, AlSi9Mg0.4, and AlSi9Mg0.6 hypoeutectic cast alloys.

The characteristic solidification temperatures were determined from the cooling curves, and their variation with Mg content is illustrated in Figure 2, Figure 3 and Figure 4. Cooling curve analysis, as illustrated in Figure 2, Figure 3 and Figure 4, revealed systematic shifts in characteristic solidification temperatures (*T*_Liq_, *T*_DCP_, *T*_Rigidity_, and *T*_Sol_) with changes in alloy composition. These shifts directly influence the evolution of dendritic networks and the permeability of the remaining liquid during solidification.

**Figure 5:**
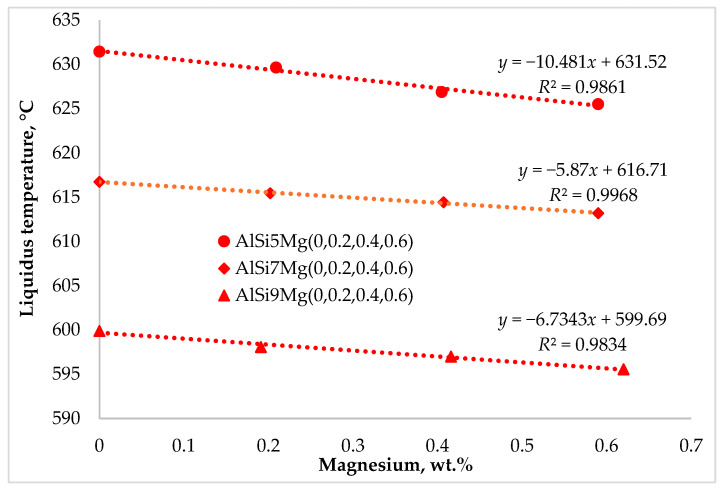
Impact of silicon and magnesium on the liquidus temperature.

As shown in Figure 5, increasing the silicon content from 5 to 9 wt.% resulted in a progressive decrease in the liquidus temperature. This behavior is consistent with the Al–Si phase diagram, which indicates that each 1 wt.% increase in silicon lowers the liquidus temperature by approximately 6.78 °C. Consequently, the variation in silicon content among the alloys investigated resulted in a reduction of about 27 °C in the overall liquidus temperature.

The addition of magnesium further reduced the liquidus temperature in all alloy series. Based on the Al–Mg phase diagram, 0.6 wt.% Mg decreased the liquidus temperature by approximately 3.3 °C. Solidification begins with the formation of primary α-Al dendrites, which reject solute elements into the remaining liquid. As solidification progresses, the liquid becomes progressively enriched in both silicon and magnesium. Increasing the silicon content promotes earlier formation of the Al–Si eutectic reaction, thereby modifying the solidification path and reducing the duration of fully liquid feeding conditions. Magnesium remains largely in solution during primary and eutectic solidification stages and contributes to Mg_2_Si formation only in the final stages of solidification at temperatures below the Al–Si eutectic reaction.

The combined effect of silicon and magnesium therefore modifies the solidification sequence and the thermal evolution of the melt. Cooling curve analysis confirmed an overall reduction of approximately 36 °C across the investigated compositions, from AlSi5 to AlSi9Mg0.6.

**Figure 6:**
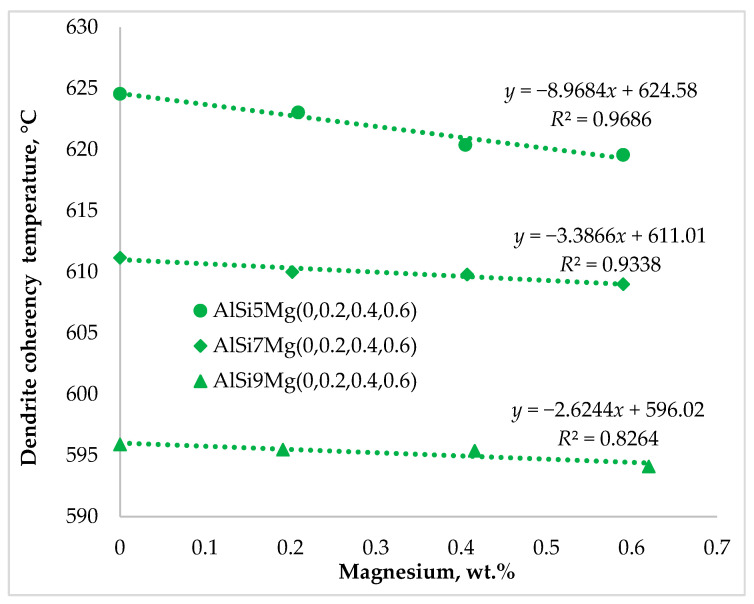
Impact of silicon and magnesium on the dendrite coherency temperature, DCT.

Figure 6 shows that the dendrite coherency temperature followed a trend similar to that of the liquidus temperature, decreasing with increasing silicon and magnesium content. The dendrite coherency temperature represents the point at which growing dendrites begin to interconnect and form a continuous network within the remaining liquid. This transition marks the end of unrestricted liquid flow and the beginning of interdendritic feeding.

Previous studies [4,6,19,20,21] have shown that both silicon and magnesium significantly influence the dendrite coherency temperature. Silicon primarily contributes to the formation of the Al–Si eutectic and alters the solidification path through solute enrichment of the interdendritic liquid, rather than directly refining dendrites. Magnesium, on the other hand, affects dendrite coherency primarily through its segregation into the interdendritic liquid during solidification, increasing solute enrichment and promoting constitutional undercooling, which facilitates earlier dendrite interlocking. In hypoeutectic Al–Si–Mg alloys, Mg_2_Si forms during the final stages of solidification from the remaining solute-enriched liquid, typically through a ternary eutectic-type reaction (*L* → *α*-Al + Si + Mg_2_Si). This transformation occurs after the primary and binary eutectic reactions and mainly affects the final solidification path and interdendritic permeability.

During the primary solidification of aluminum alloys, solute partitioning at the solid–liquid interface enriches the interdendritic liquid with alloying elements. The resulting constitutional undercooling promotes dendritic growth and strongly influences dendrite arm spacing, thereby affecting the temperature at which dendritic networks become mechanically coherent. Magnesium remains largely in solid solution during the early stages of solidification; however, its concentration in the remaining liquid progressively increases due to segregation. This enrichment alters the thermophysical properties of the interdendritic melt and modifies the final stages of solidification.

Magnesium addition promotes earlier dendrite interlocking primarily through solute enrichment of the interdendritic liquid and enhanced constitutional undercooling. At the same time, Mg_2_Si formation occurs at later stages of solidification and does not directly influence the onset of dendrite coherency [18,22]. The diagram indicates a DCT decrease of approximately 5 °C for Series 1 alloys (5 wt.% Si), whereas Series 2 (7 wt.% Si) and Series 3 (9 wt.% Si) exhibited smaller reductions of about 2 °C. These changes directly influence the transition from mass feeding to interdendritic feeding.

**Figure 7:**
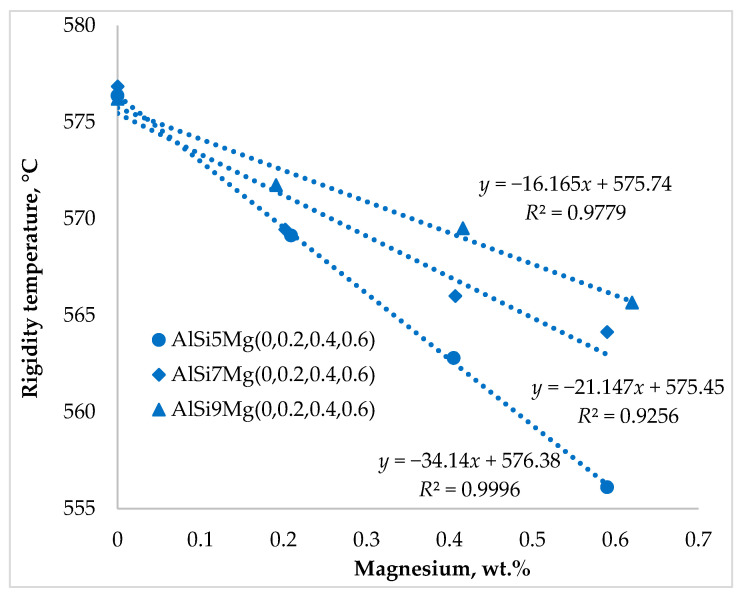
Impact of silicon and magnesium on the rigidity temperature.

Figure 7 illustrates the variation in rigidity temperature with alloy composition. In contrast to silicon, which produced only minor changes in rigidity temperature, magnesium addition caused a pronounced decrease in this characteristic temperature across all alloy series. The largest reduction was observed in alloys containing 5 wt.% Si, where increasing magnesium content significantly lowered the rigidity temperature.

**Figure 8:**
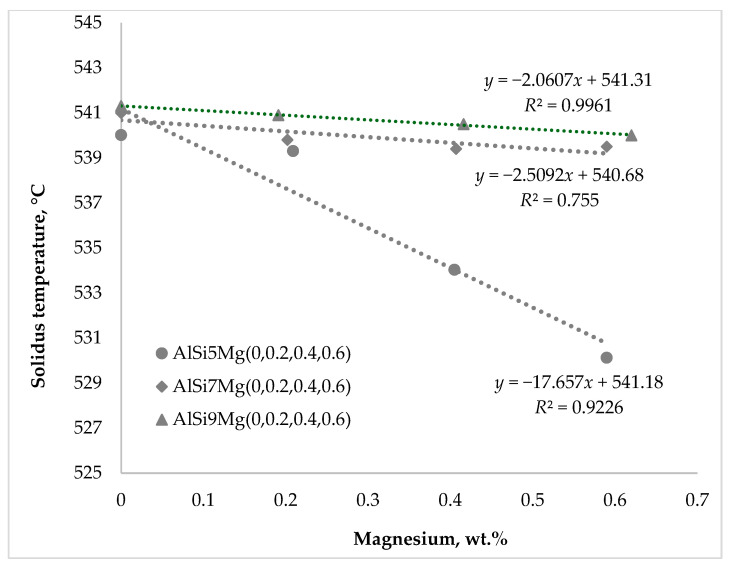
Impact of silicon and magnesium on the solidus temperature.

The rigidity temperature represents the point at which the dendritic network becomes sufficiently rigid to resist deformation, resulting in a substantial reduction in melt permeability. At this stage, interdendritic channels become narrow and liquid feeding becomes increasingly difficult. The incorporation of magnesium therefore modifies dendritic growth and microstructural morphology, influencing both the permeability of the dendritic network and the efficiency of liquid feeding. A similar trend was observed for the solidus temperature in Figure 8.

The addition of magnesium decreased the solidus temperature in all alloy series, with the most pronounced reduction of approximately 10 °C occurring in the alloys containing 5 wt.% Si. The results presented in Figure 7 and Figure 8 demonstrate that variations in magnesium content exerted a considerably stronger influence on rigidity and solidus temperatures than comparable changes in silicon content. This indicates that magnesium plays a dominant role in controlling the late stages of solidification and the final feeding conditions in hypoeutectic Al–Si–Mg alloys.

**Figure 9:**
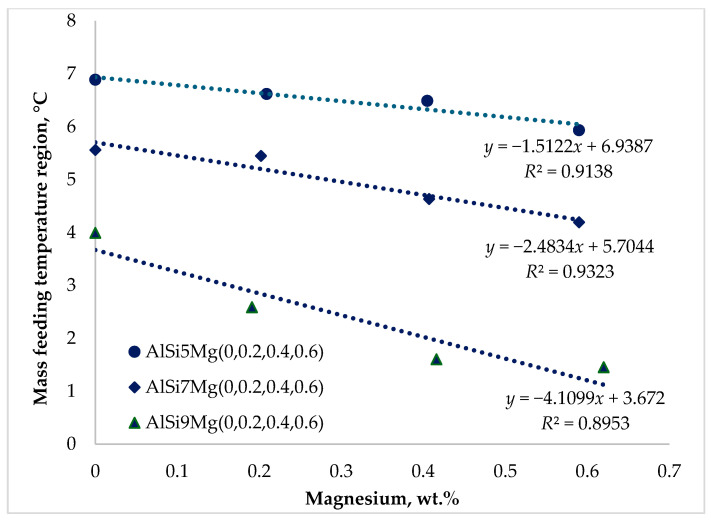
Impact of silicon and magnesium on the mass feeding (*MF*) region.

**Figure 10:**
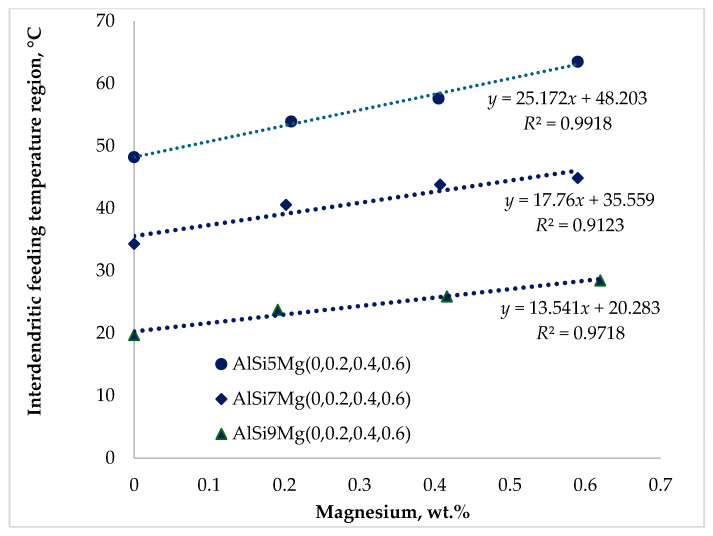
Impact of silicon and magnesium on the interdendritic feeding (*IDF*) region.

**Figure 11:**
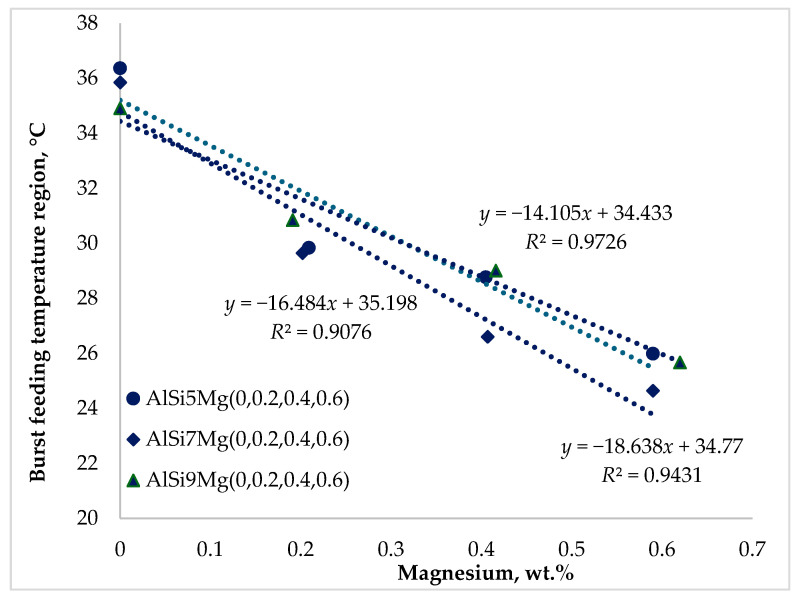
Impact of silicon and magnesium on the burst feeding (*BF*) region.

It is widely reported in the literature that feeding behavior during the solidification of aluminum casting alloys is strongly influenced by chemical composition [23,24,25,26,27,28,29,30]. The results presented in Figure 9, Figure 10 and Figure 11 demonstrate that both silicon and magnesium contents significantly affect the feeding temperature ranges of the investigated alloys.

Increasing the silicon and magnesium contents reduces the temperature ranges associated with mass and burst feeding, while simultaneously expanding the temperature interval for interdendritic feeding. This shift indicates that feeding progressively occurs at lower temperatures and higher solid fractions as the alloying content increases.

Figure 9 shows that the mass-feeding temperature range decreased moderately with increasing magnesium content. The most pronounced reduction occurred in the Series 3 alloys containing 9 wt.% Si, where increasing magnesium from 0.2 to 0.6 wt.% reduced the mass-feeding range by approximately 2.5 °C. For Series 1 and Series 2 alloys, the reductions were smaller, amounting to approximately 0.95 °C and 1.37 °C, respectively.

The mass-feeding region occurs at the early stage of solidification, when the fraction of solid α-Al is still relatively low, and the melt viscosity remains low. Under these conditions, liquid metal can easily flow through the casting cavity, and feeding channels remain wide and unobstructed. Consequently, variations in alloy composition have only a limited influence on feeding behavior during this stage.

Figure 10 illustrates a substantial expansion of the interdendritic feeding temperature (*IDF*) range with increasing magnesium content. The increases were approximately 15.22 °C for Series 1, 10.54 °C for Series 2, and 8.74 °C for Series 3 alloys. Increasing the silicon content from 5 to 9 wt.% partially moderated this expansion; however, magnesium addition still produced a significant widening of the interdendritic feeding interval.

The interdendritic feeding region is defined by two critical temperatures: the dendrite coherency temperature and the rigidity temperature. Between these two temperatures, the dendritic network has already formed but remains partially permeable, allowing liquid metal to flow through narrow interdendritic channels. As the temperature decreases, the solid fraction increases, and the remaining liquid becomes increasingly confined within these channels.

A wider interdendritic feeding interval therefore corresponds to feeding occurring at lower temperatures, higher solid fractions, and increased melt viscosity. These conditions significantly reduce the melt permeability, making liquid feeding more difficult. Consequently, an expansion of this temperature range is generally considered unfavorable because it increases the susceptibility to the formation of shrinkage porosity. The proposed relationship between magnesium addition, redistribution of feeding regions, and increased porosity should be interpreted in the context of established solidification theory. While the present study did not include the direct microstructural characterization of Mg_2_Si, the observed expansion of the interdendritic feeding region indicates reduced permeability and restricted liquid flow at higher solid fractions. Based on the literature [9,18,25,26], Mg segregation and subsequent Mg_2_Si formation during the final stages of solidification are expected to limit interdendritic feeding further. Therefore, the mechanism proposed here represents a physically supported interpretation of the observed correlations rather than direct experimental verification.

The observed increase in interdendritic feeding and associated porosity is consistent with previous studies on permeability reduction at higher solid fractions. However, unlike most literature, which typically considers the influence of individual alloying elements, the present results provide a quantitative description of how the combined effect of Si and Mg redistributes feeding regions during solidification.

Figure 11 shows that magnesium addition had a significantly stronger influence on burst feeding than silicon. Increasing the magnesium content consistently reduced the burst-feeding temperature range across all alloy series. The reduction was approximately 10.4 °C for Series 1, 11.2 °C for Series 2, and 9.3 °C for Series 3 alloys.

The rigidity temperature marks the transition from interdendritic feeding to burst feeding. Below this temperature, feeding occurs only by gravity or by the hydrostatic pressure of the remaining liquid metal. Since the dendritic network has already become rigid, liquid flow through interdendritic channels is severely restricted. The observed reduction of the burst feeding interval therefore reflects the strong influence of magnesium on the final stages of solidification.

To quantitatively describe the relative contributions of the different feeding mechanisms, temperature ratios were calculated using the following expressions [26]:

(1) $$MF=\frac{T_{LIQ}-T_{DCP}}{T_{LIQ}-T_{SOL}}\times 100$$

(2) $$IDF=\frac{T_{DCP}-T_{Rigidity}}{T_{LIQ}-T_{SOL}}\times 100$$

(3) $$BF=\frac{T_{Rigidity}-T_{SOL}}{T_{LIQ}-T_{SOL}}\times 100$$

where *MF*, *IDF*, and *BF* represent the temperature ratios corresponding to mass feeding, interdendritic feeding, and burst feeding, respectively. The temperature ratio approach provides a normalized metric independent of the absolute solidification range, enabling a direct comparison of feeding redistribution among alloys with different compositions.

**Figure 12:**
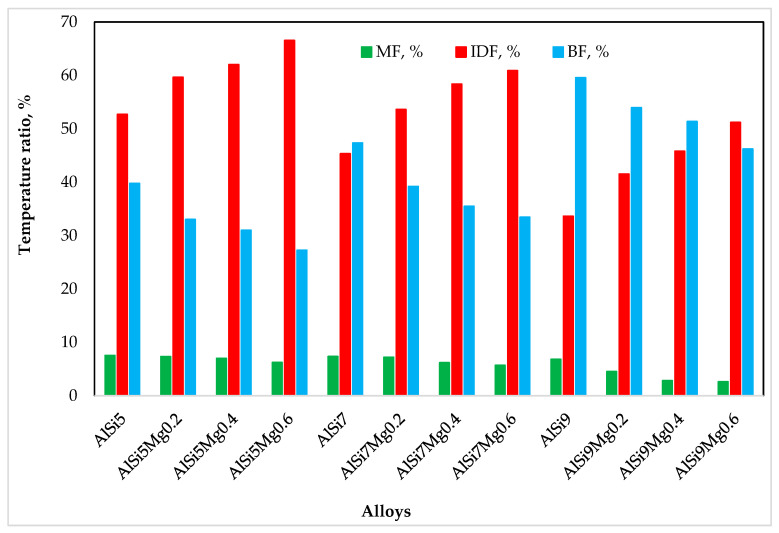
The impact of silicon and magnesium on the temperature ratio of different feeding regions: mass feeding (*MF*), interdendritic feeding (*IDF*), and burst feeding (*BF*) regions.

Applying Equations (1)–(3) and calculating the corresponding temperature ratios for various feeding regions, the impact of silicon and magnesium was quantified and is presented in Figure 12.

Figure 12 presents the redistribution of feeding contributions among the three feeding mechanisms for all alloy series investigated. For alloys containing 5 wt.% Si, the temperature ratio of interdendritic feeding increased markedly with magnesium addition, rising from approximately 53% to about 67% as the magnesium content increased from 0 to 0.6 wt.%. At the same time, the burst-feeding ratio decreased from about 40% to approximately 27%, while the mass-feeding ratio decreased slightly from roughly 9% to about 7%.

A similar but somewhat less pronounced trend was observed in the alloys containing 7 wt.% Si, where the interdendritic feeding ratio increased from about 45% to approximately 61% with increasing magnesium content. In contrast, the alloys containing 9 wt.% Si initially exhibited dominant burst feeding in the magnesium-free condition. However, with increasing magnesium content, the feeding balance again shifted toward interdendritic feeding.

Overall, these results clearly demonstrate that magnesium plays a dominant role in redistributing feeding mechanisms toward interdendritic-controlled flow during solidification.

The practical implications of this redistribution were evaluated using the Sand Hourglass test.

**Figure 13:**
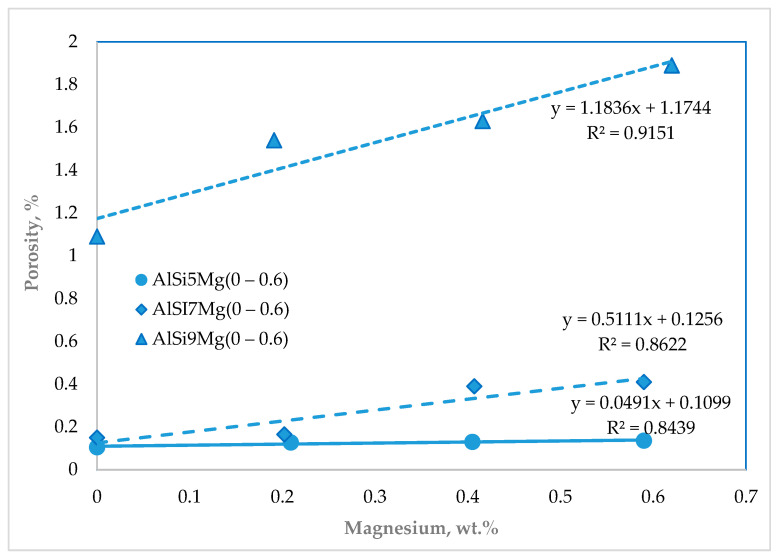
Impact of silicon and magnesium on the porosity.

Image analysis of the lower regions of the Sand Hourglass samples confirmed that porosity increased with increasing magnesium content across all alloy series. Alloys containing 5 and 7 wt.% Si without magnesium exhibited very low porosity levels of approximately 0.1%. However, increasing the silicon content to 9 wt.% resulted in a higher porosity level of about 1%, Figure 13.

The addition of magnesium further increased the porosity in all alloy series, with the highest value of approximately 1.9% recorded for the AlSi9Mg0.6 alloy. These results clearly indicate that magnesium addition significantly increases the alloys’ susceptibility to shrinkage porosity.

**Figure 14:**
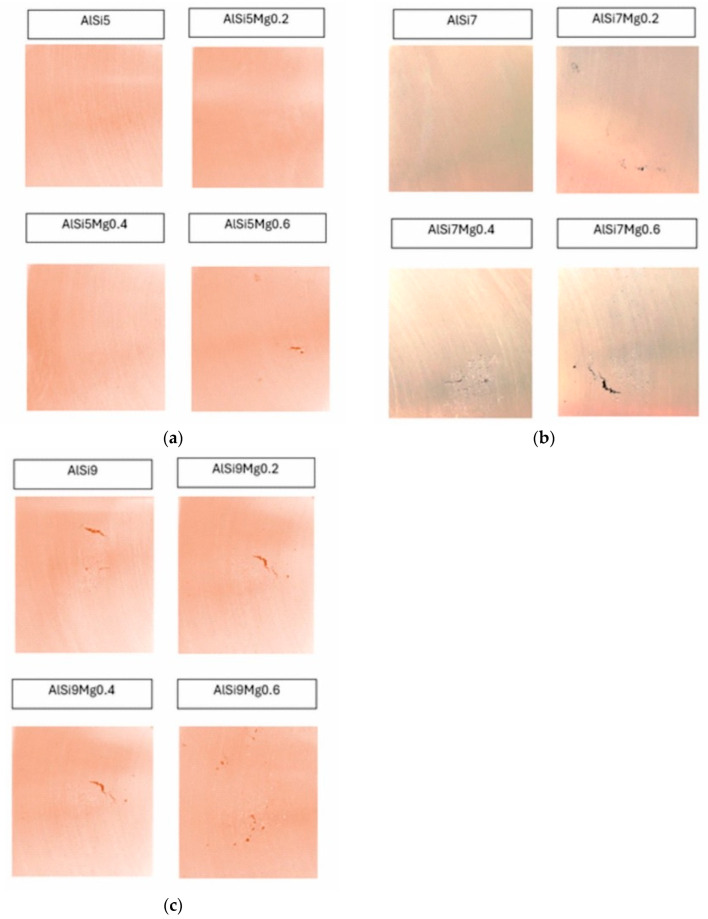
Photograph of metallographic Sand Hourglass test samples for alloys series 1 (**a**), for alloys series 2 (**b**) [18], and for alloys series 3 (**c**). The images represent polished cross-sections from the central region of the lower specimen. The color contrast is due to surface preparation and lighting conditions and does not reflect the alloy’s actual color.

The observed increase in porosity can be explained by changes in the interdendritic region caused by magnesium addition, Figure 14. As magnesium segregates during solidification, it contributes to the formation of Mg-rich intermetallic phases and modifies the morphology of interdendritic channels. The resulting reduction in channel width restricts liquid flow and reduces feeding efficiency during the final stages of solidification, ultimately promoting the formation of shrinkage porosity in the investigated alloys.

These microstructural changes can be directly linked to magnesium’s compositional influence on solidification dynamics and interdendritic flow resistance. As the magnesium content increases, the redistribution of feeding paths becomes more pronounced, leading to localized feeding limitations and a greater tendency for shrinkage-related defects. These results confirm that even moderate compositional modifications can significantly alter feeding conditions during the solidification of hypoeutectic Al–Si–Mg alloys.

## 4. Conclusions

This study investigated the influence of silicon and magnesium on the solidification behavior and feeding effectiveness of hypoeutectic Al–Si–Mg alloys using cooling curve analysis and the Sand Hourglass test. Based on the results obtained, the following conclusions can be drawn:

1. Increasing the silicon content from 5 to 9 wt.% and magnesium content from 0 to 0.6 wt.% systematically reduced the liquidus, dendrite coherency, rigidity, and solidity temperatures, thereby modifying the overall solidification sequence of the investigated alloys.
2. Magnesium exhibited a significantly stronger influence on the reduction in rigidity and solidus temperatures than silicon, indicating that Mg primarily affects the late stages of solidification.
3. Increasing silicon and magnesium contents led to a redistribution of feeding mechanisms during solidification. The temperature ranges of mass feeding and burst feeding decreased, while the interdendritic feeding range expanded.
4. Temperature ratio analysis confirmed that magnesium addition progressively shifts the feeding mechanism toward interdendritic feeding, for example, in alloys containing 5 wt.% Si, the interdendritic feeding ratio increased from approximately 53% to 67% as the magnesium content increased from 0 to 0.6 wt.%, while the burst feeding contribution decreased accordingly.
5. The expansion of the interdendritic feeding region corresponds to more restricted liquid flow through the dendritic network, resulting in less efficient feeding conditions.
6. Sand Hourglass tests confirmed that increasing magnesium content significantly increased the shrinkage porosity in all alloy series. The highest porosity level (~1.9%) was observed in the AlSi9Mg0.6 alloy.
7. The increased susceptibility to shrinkage porosity is primarily attributed to interdendritic channel narrowing and the formation of Mg_2_Si phases, which obstruct feeding paths and reduce liquid feeding capability during the final stages of solidification.

Overall, the results demonstrate that magnesium plays a dominant role in controlling feeding redistribution and porosity formation in hypoeutectic Al–Si–Mg alloys. Understanding these compositional effects provides an important basis for optimizing alloy design and casting parameters to minimize shrinkage defects in aluminum castings. These findings highlight the importance of the composition-based control of solidification dynamics to improve feeding efficiency and reduce shrinkage susceptibility in aluminum casting design.

## Figures and Tables

**Table 1:** Chemical composition of the prepared AlSi(5, 7, 9)Mg(0, 0.2, 0.4, 0.6) alloys.

Alloy	Si, wt.%	Mg, wt.%	Cu, wt.%	Mn, wt.%	Cr, wt.%	Ni, wt.%	Zn, wt.%
AlSi5	4.96	0	0.007	0.02	0.001	0.001	0.006
AlSi5Mg0.2	4.86	0.209	0.008	0.003	0.002	0.002	0.007
AlSi5Mg0.4	5.11	0.405	0.007	0.004	0.001	0.002	0.008
AlSi5Mg0.6	4.84	0.590	0.021	0.004	0.002	0.001	0.008
AlSi7	6.8	0	0.004	0.03	0.001	0.001	0.006
AlSi7Mg0.2	6.92	0.202	0.007	0.004	0.002	0.002	0.008
AlSi7Mg0.4	6.81	0.407	0.007	0.005	0.002	0.002	0.009
AlSi7Mg0.6	6.81	0.59	0.006	0.004	0.002	0.001	0.008
AlSi9	8.8	0	0.006	0.015	0.001	0.001	0.005
AlSi9Mg0.2	8.82	0.191	0.008	0.002	0.001	0.001	0.008
AlSi9Mg0.4	9.03	0.416	0.011	0.003	0.003	0.003	0.006
AlSi9Mg0.6	8.75	0.62	0.007	0.003	0.002	0.002	0.006

## Data Availability

The original contributions presented in this study are included in the article/Supplementary Materials. Further inquiries can be directed to the corresponding author.

## Supplementary Materials

The following supporting information can be downloaded at: https://www.mdpi.com/article/10.3390/ma19091744/s1, Minimal dataset.
